# Stereotactic Radiosurgery for an Unresectable Jugulotympanic Paraganglioma: Clinical Response and Acute Toxicity in a 70-Year-Old Patient

**DOI:** 10.7759/cureus.116729

**Published:** 2026-09-22

**Authors:** Karla Valeria Hernandez Barragan, Yesenia Ortega Flores, Arizbeth Arredondo Alvarez, Cruz Alejandro Valdivia Esquivel

**Affiliations:** 1 Otolaryngology - Head and Neck Surgery, Centenario Hospital Miguel Hidalgo, Aguascalientes, MEX; 2 Radiation Oncology, Centenario Hospital Miguel Hidalgo, Aguascalientes, MEX

**Keywords:** glomus jugulare, glomus tympanicum, photon stereotactic radiosurgery, pulsatile tinnitus, radiation side effects, skull base tumors, stereotactic ablative radiation

## Abstract

Jugulotympanic paragangliomas (JTPs) are rare, highly vascular neuroendocrine tumors characterized by slow but locally aggressive growth at the skull base. Their management in elderly patients is particularly challenging because of their close proximity to critical neurovascular structures. We report the case of a 70-year-old woman with a 20-year history of pulsatile tinnitus and a 13-year history of chronic vertigo. Her symptoms progressively worsened, with the recent onset of otic bleeding, occipital headache, and dysphonia, prompting referral to the Radiation Oncology Department. Physical examination revealed a pulsatile reddish mass in the left middle ear, along with ipsilateral lower cranial nerve deficits. Given the unresectable nature of the lesion and the patient’s comorbidities, definitive stereotactic radiosurgery (SRS) was recommended. Treatment was delivered in a single fraction of 14 Gy with the aim of achieving local tumor control and symptom stabilization. Following treatment, the patient experienced significant improvement in pulsatile tinnitus and complete resolution of occipital pain. At the three-month follow-up evaluation, she developed local complications including tympanic membrane ulceration and middle ear mucosal necrosis, which were managed conservatively with antibiotic therapy and close clinical surveillance. At eight months after treatment, the patient maintained clinical improvement and neurological stability, with persistent local tumor control on follow-up MRI and no evidence of locoregional progression. SRS represents an effective treatment option for unresectable JTPs, offering symptom control and functional preservation with acceptable toxicity. Nevertheless, careful post-treatment monitoring is essential for the early identification and management of radiation-related complications, particularly in tumors with extensive local invasion.

## Introduction

Jugulotympanic paragangliomas (JTPs) are rare neuroendocrine neoplasms characterized by locally aggressive behavior, arising from the paraganglionic tissue of the jugular bulb and middle ear [1]. These tumors are highly hypervascular and exhibit slow but progressive growth, with a marked capacity for osteolytic destruction of the temporal bone [1,2]. As they enlarge, they progressively involve the lower cranial nerves (IX-XII), typically presenting with pulsatile tinnitus, hearing loss, and severe neurological morbidity, including dysphagia and dysphonia [3,4]. Epidemiologically, JTPs occur predominantly in women, with a female-to-male ratio ranging from 3:1 to 6:1, and are most frequently diagnosed between the fifth and seventh decades of life. Notably, up to 40% of cases are associated with hereditary syndromes linked to mutations in the succinate dehydrogenase (SDH) genes [5,6]. In older adults, their indolent clinical course frequently delays diagnosis, allowing continued anatomical progression that adversely affects long-term functional outcomes [6-8].

The management of unresectable or locally advanced JTPs remains a significant therapeutic challenge. Their extensive vascularity and intimate relationship with critical neurovascular structures often render surgical resection technically demanding and associated with substantial risks of morbidity and mortality [9,10]. Consequently, the therapeutic paradigm has shifted from radical surgical excision toward functional preservation, with radiation oncology assuming a central role in disease management [9,11].

External beam radiotherapy (EBRT) and other highly conformal radiation techniques constitute the cornerstone of definitive treatment for inoperable tumors and are also used in the adjuvant setting. From a radiobiological perspective, radiation therapy achieves durable tumor control primarily by suppressing cellular proliferation and differentiation while inducing progressive microvascular sclerosis and stromal fibrosis [12,13]. Using conventionally fractionated regimens (45-60 Gy), EBRT has demonstrated response rates ranging from 70% to 100% and long-term local control rates of 78%-93% [12,13].

More recently, stereotactic radiation techniques - including stereotactic radiosurgery (SRS), stereotactic body radiotherapy (SBRT), and fractionated stereotactic radiotherapy (FSRT) - have transformed the management of JTPs. Nevertheless, their use requires careful monitoring for both acute toxicities, such as mucositis, dermatitis, and fatigue, and late adverse effects, including radionecrosis, cranial neuropathies, sensorineural hearing loss, and radiation-induced meningiomas [8]. These highly precise modalities provide excellent anatomical stabilization and substantial symptomatic improvement while maximizing the sparing of adjacent organs at risk (OARs) and reducing treatment-related morbidity compared with surgery [14].

In this context, we present the clinical findings and longitudinal course of an unresectable JTP treated with SRS. The aim of this report is to document the effectiveness of SRS in achieving local tumor control while highlighting specific acute radiation-related complications, including tympanic ulceration and middle ear mucosal necrosis. By describing the patient's long-term clinical and radiographic response, this case contributes additional evidence regarding the role of SRS in the management of skull base tumors and underscores the importance of close neuro-oncologic follow-up after treatment.

## Case presentation

We report the case of a 70-year-old woman from Aguascalientes, Mexico. Her medical history was significant for type 2 diabetes mellitus (T2DM) with a positive family history, systemic arterial hypertension, dyslipidemia, and osteoporosis. Her social history included occasional alcohol consumption (approximately five drinks per month) and a previous SARS-CoV-2 infection.

The patient's symptoms had begun approximately 20 years before presentation, initially manifesting as left-sided tinnitus associated with ipsilateral occipital headache. She subsequently developed vertigo, which had been present for approximately 13 years. Two weeks before her initial evaluation, both the headache and vertigo worsened significantly. During the same period, she experienced an episode of left-sided otorrhagia that required hospital admission for observation; however, blood transfusion was not necessary.

Physical examination by the Radiation Oncology service revealed a reddish, pulsatile mass visible within the left middle ear on otoscopic examination. Neurological examination demonstrated left lower cranial nerve deficits and dysphonia, suggesting tumor extension toward the jugular foramen with involvement of adjacent critical neurovascular structures.

The extent and anatomical characteristics of the lesion were evaluated by contrast-enhanced magnetic resonance imaging (MRI), which demonstrated a left JTP involving critical skull base structures. The lesion is shown in the axial plane in Figure 1A and the coronal plane in Figure 1B. The tumor was considered unresectable because radical surgical resection was associated with an unacceptably high risk of functional morbidity. Given the unresectable nature of the lesion, its intimate relationship with critical skull base neurovascular structures, and the patient's age and comorbidities, definitive SRS was selected as the preferred treatment modality.

**Figure 1:**
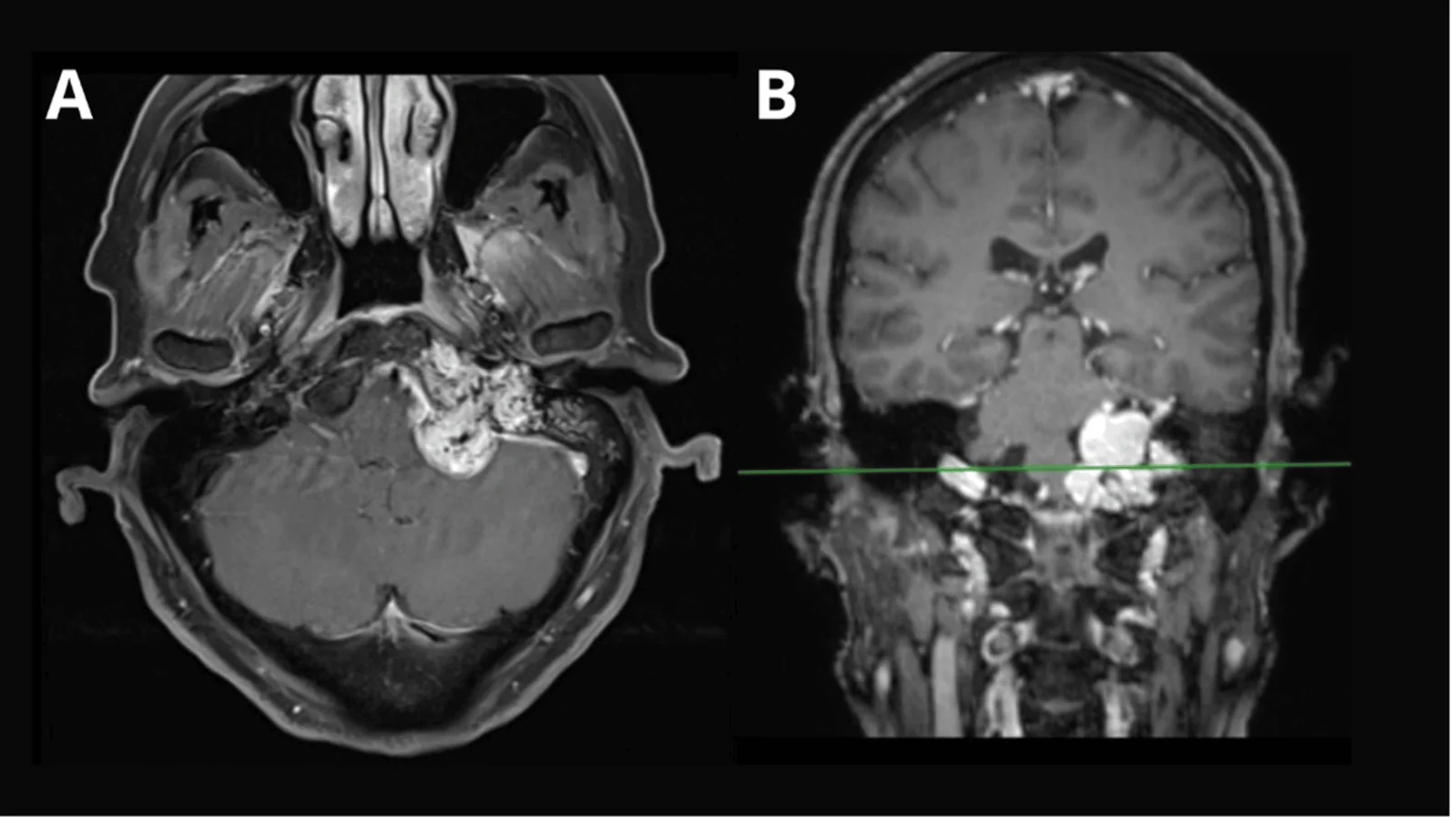
Basal MRI Contrast-enhanced brain magnetic resonance imaging (MRI). Axial (A) and coronal (B) images demonstrate a heterogeneous, intensely enhancing, lobulated, extra-axial, expansile lesion in the left petroclival skull base. Medial mass effect on the brainstem and adjacent cerebellar structures is evident.

Treatment was delivered using a single-fraction radiosurgical approach with a prescription dose of 14 Gy. The planning target volume (PTV) was generated by applying a 0.5-mm isotropic expansion to the gross tumor volume delineated on contrast-enhanced MRI. Treatment planning was performed using the Eclipse™ Treatment Planning System, version 16.1 (Varian Medical Systems, Palo Alto, CA, USA), at Centenario Hospital Miguel Hidalgo. Dosimetric planning prioritized optimal target coverage while maintaining strict OAR constraints to maximize local tumor control and minimize treatment-related toxicity. The treatment plan was designed to achieve coverage of more than 98% of the PTV by the 12-Gy isodose line (V12 Gy > 98%) while maintaining a maximum dose below 180% of the prescription dose. Additional constraints included a maximum dose to healthy brain tissue of <16 Gy, brain V12 Gy <10 cc, maximum brainstem dose <12 Gy, mean parotid dose <4 Gy, mean pharyngeal dose <8 Gy, and maximum spinal cord dose <10 Gy.

The final treatment plan complied with all predefined dosimetric constraints. The maximum dose to the brainstem was 10.6 Gy, while the mean dose to the left parotid gland was 3.22 Gy, the mean dose to the pharynx was 3.83 Gy, and the maximum dose to the spinal cord was 6.72 Gy. SRS was delivered using volumetric modulated arc therapy (VMAT) with two coplanar arcs in clockwise and counterclockwise rotation, with a total of 8,364 monitor units. Treatment was performed on a Varian® VitalBeam™ linear accelerator (Varian Medical Systems, Palo Alto, California, USA) equipped with a Millennium multileaf collimator and a PerfectPitch™ (Varian Medical Systems) six-degree-of-freedom treatment couch. Image-guided verification was performed using cone-beam computed tomography (CBCT) immediately before treatment delivery. The primary treatment objectives were durable local tumor control, symptom stabilization, and preservation of neurological function. During the early post-treatment period, the patient developed mild left preauricular pain and localized soft-tissue swelling, which resolved completely after a single dose of etoricoxib.

At the three-month follow-up evaluation, the patient demonstrated significant clinical improvement. Occipital pain had completely resolved, and pulsatile tinnitus had markedly decreased compared with the pretreatment baseline. No new neurological deficits were identified. However, she subsequently developed approximately one week of left-sided otorrhea. Otoscopic examination revealed ulceration of the tympanic membrane and areas of necrosis involving the middle ear mucosa. Antibiotic therapy with cefixime was initiated, together with close clinical surveillance. Based on the clinical presentation and the requirement for medical intervention without invasive treatment or hospitalization, this treatment-related toxicity was retrospectively classified as grade 2 according to the Common Terminology Criteria for Adverse Events (CTCAE), version 5.0.

At eight months after SRS, the middle ear mucosal necrosis had completely resolved on otoscopic examination, with restoration of the local mucosal appearance. Neurological examination also demonstrated functional improvement, with greater symmetry of the tongue and reduced tongue deviation compared with the pretreatment examination. Bilateral trapezius muscle strength was preserved at 5/5. Follow-up contrast-enhanced MRI demonstrated a slight reduction in tumor dimensions, measuring 31 × 31 × 38 mm compared with 31 × 31 × 40 mm at baseline. Although this degree of dimensional change did not meet the Response Evaluation Criteria in Solid Tumors (RECIST) 1.1 criteria for partial response, a substantial reduction in contrast enhancement was observed, with no evidence of locoregional progression or new lesions, supporting stable disease with early post-radiosurgical treatment-related changes (Figure 2). The lesion is shown in the axial plane in Figure 2A and the coronal plane in Figure 2B. An additional quantitative finding was observed on diffusion-weighted imaging and apparent diffusion coefficient (ADC) mapping. The ADC value within the lesion increased from 1.198 × 10⁻³ mm²/s on the previous MRI to 1.330 × 10⁻³ mm²/s at eight months after SRS. This increase in ADC, together with the reduction in contrast enhancement and tumor volume, was interpreted as a complementary imaging finding suggestive of decreased tumor cellularity and treatment-related tissue injury, potentially reflecting progressive tumor necrosis. Longitudinal MRI surveillance was planned to further evaluate volumetric response, diffusion characteristics, and the structural integrity of the adjacent skull base structures.

**Figure 2:**
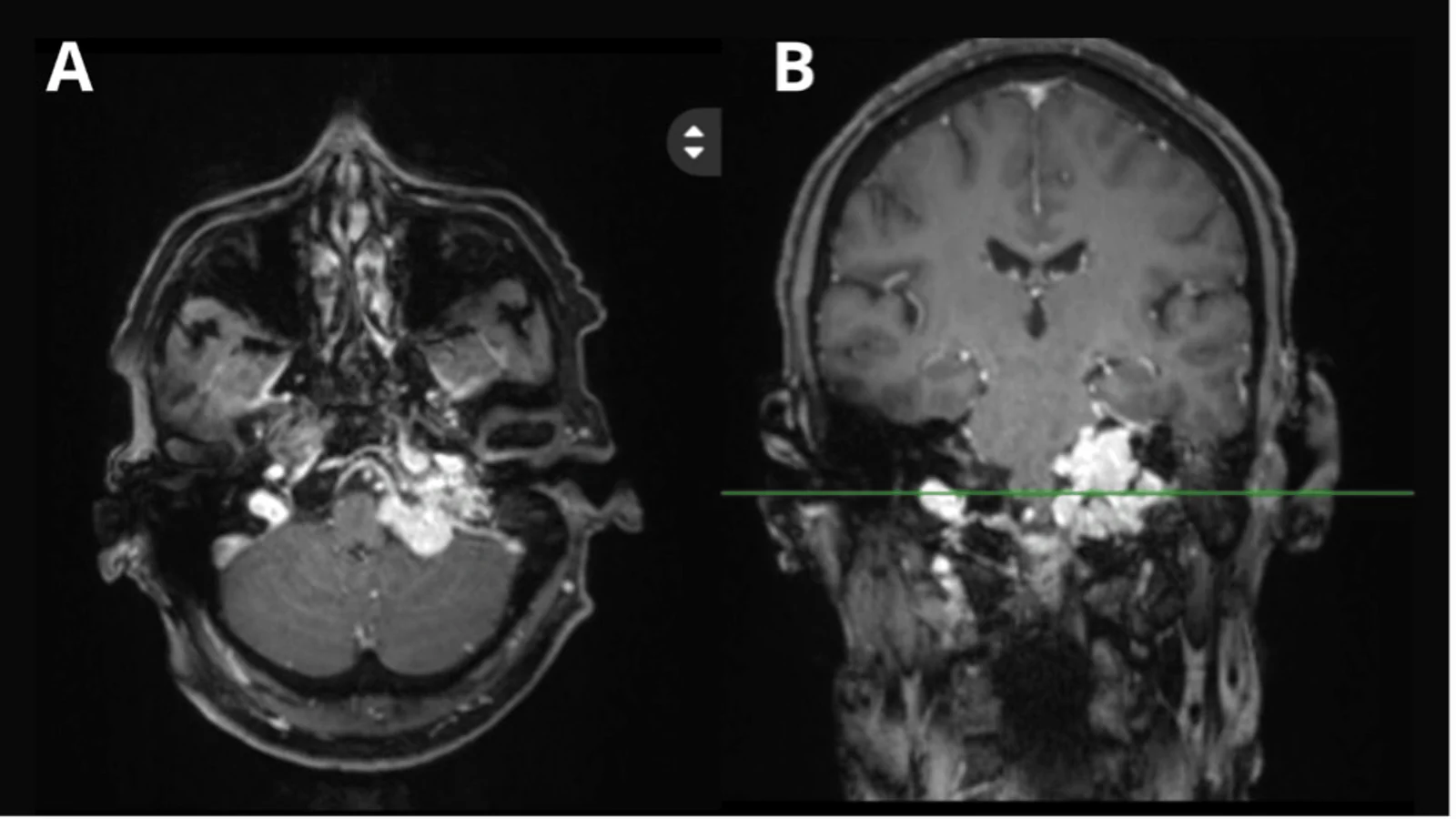
Contrast-enhanced brain magnetic resonance imaging (MRI) at eight months of follow-up Axial (A) and coronal (B) images demonstrate the previously described extra-axial lesion in the left petroclival region, which continues to show intense enhancement following gadolinium administration. Compared with the baseline study, there is a reduction in tumor volume and mass effect on the brainstem and adjacent structures, with no evidence of new lesions or locoregional progression. For the management of these findings, treatment with cefixime and mosapride was prescribed, and follow-up MRI was scheduled for 2026 to assess tumor volumetric response and the integrity of adjacent structures.

## Discussion

The multidisciplinary evaluation of this case, integrating the perspectives of Otolaryngology, Skull Base Surgery, and Radiation Oncology, highlights the inherent complexity of managing JTPs involving critical skull base anatomy [1,7]. Given the locally invasive behavior and distinct molecular and genetic profiles of these tumors [6], current treatment strategies prioritize durable local tumor control while preserving neurological function and maintaining quality of life [5].

Historically, surgical resection represented the standard of care. However, in advanced lesions (Fisch class C or D), surgery is associated with an unacceptably high incidence of permanent lower cranial nerve (IX-XII) deficits [8,10]. Previous studies have shown that radical surgery approximately doubles the risk of severe neurological complications, including disabling dysphagia requiring gastrostomy, compared with radiation-based treatment modalities [4,13]. Furthermore, in this 70-year-old patient with multiple systemic comorbidities, including T2DM and hypertension, the perioperative risks associated with the marked hypervascularity of the tumor - particularly massive hemorrhage and ischemic complications - were considered prohibitive, limiting the feasibility of extensive skull base resection or aggressive preoperative embolization [11,12]. Although active surveillance ("wait-and-scan") remains an acceptable strategy for asymptomatic lesions, the presence of otorrhagia and worsening vertigo in our patient indicated biologically active disease requiring definitive treatment to halt further progression and reduce the risk of additional neurovascular complications [1].

Based on these considerations, SRS was selected as the treatment of choice. A marginal dose of 14 Gy prescribed to the PTV was chosen after careful consideration of the therapeutic ratio. Multiple meta-analyses have demonstrated that marginal doses between 14 and 20 Gy provide long-term local control rates exceeding 90%-95% in patients with JTPs [2,13,15,16]. In the present case, the lower end of this therapeutic range was selected because of the close proximity of the target volume to critical OARs, particularly the brainstem, while also aiming to minimize radiation-related toxicity in tissues potentially compromised by diabetes- and hypertension-associated microvascular disease.

From a radiobiological standpoint, the efficacy of conformal radiotherapy and SRS in JTPs is not primarily attributable to immediate cytoreduction but rather to progressive vascular injury within the tumor. Ionizing radiation induces endothelial senescence and apoptosis, leading to progressive obliterative microvascular sclerosis and extensive stromal fibrosis [17-19]. This progressive loss of tumor perfusion arrests tumor growth and explains the gradual improvement in symptoms observed after treatment. Comparative series using 15 Gy have reported symptomatic improvement in up to 47% of patients [13], consistent with the marked reduction in pulsatile tinnitus experienced by our patient three months after SRS.

The pathophysiology underlying the acute middle ear toxicity observed in this case is likely multifactorial. Tumor extension into the middle ear cavity increased the volume of irradiated mucosa, predisposing the patient to early radiation-induced injury [15,16]. At the molecular level, ionizing radiation induces DNA double-strand breaks in basal epithelial cells, activating transcription factors such as nuclear factor-kappa B (NF-κB). This process initiates a cascade of pro-inflammatory cytokines - including interleukin-6 (IL-6), interleukin-10 (IL-10), and tumor necrosis factor-alpha (TNF-α) - together with ceramide- and caspase-mediated signaling pathways that promote the expression of pro-apoptotic genes [17]. The resulting epithelial cell loss ultimately manifests as tympanic membrane ulceration and middle ear mucosal necrosis [16].

Although radiation-induced mucositis during conventionally fractionated radiotherapy generally becomes clinically apparent only after cumulative doses exceeding approximately 30 Gy, the substantially higher biologically effective dose (BED) delivered by single-fraction SRS accelerates these tissue responses despite the lower physical dose. Once mucosal integrity is compromised, devitalized tissue with impaired microvascular perfusion becomes highly susceptible to colonization by commensal bacteria and opportunistic pathogens, particularly *Pseudomonas aeruginosa*, thereby increasing the risk of necrotizing otitis [3]. In this context, antibiotic therapy played a critical prophylactic and therapeutic role in preventing secondary infection and subsequent osteoradionecrosis. Formal audiometry was not performed because no baseline audiometric assessment was available at the time of treatment. The authors prioritized the diagnostic and monitoring procedures considered essential for treatment planning and clinical surveillance, while avoiding additional nonessential investigations in the absence of a baseline audiometric study.

Post-radiotherapy follow-up requires advanced imaging protocols, particularly MRI, to distinguish radiation-induced exudative and inflammatory changes from true tumor progression [14]. Neuroradiological assessment, based on Response Assessment in Neuro-Oncology (RANO)-like criteria, indicates that acute treatment-related changes typically peak during the first few weeks after treatment, whereas definitive volumetric and radiographic stabilization is optimally assessed between three and six months after the intervention. Long-term clinical and imaging surveillance is therefore essential [9]. Diffusion-weighted imaging and ADC mapping may provide complementary information regarding tissue cellularity and treatment response. In the present case, the most significant longitudinal radiological change was a quantitative increase in the ADC value within the lesion, from 1.198 × 10⁻³ mm²/s on the previous MRI to 1.330 × 10⁻³ mm²/s on the eight-month follow-up study. This increase in ADC suggests a reduction in tumor cellularity and increased extracellular water mobility, findings that may be consistent with treatment-related tissue injury and tumor necrosis. These diffusion changes, together with the observed reduction in tumor volume and mass effect and the absence of new lesions or locoregional progression, support a favorable radiological response to SRS.

## Conclusions

SRS may represent a useful treatment option for selected patients with unresectable JTPs, particularly when preservation of neurological function and avoidance of extensive skull-base surgery are important considerations. In the present case, single-fraction SRS with 14 Gy was associated with substantial symptomatic improvement, including complete resolution of occipital pain and marked reduction in long-standing pulsatile tinnitus. At eight months of follow-up, the patient maintained clinical improvement, with neurological stability and improvement in selected cranial nerve findings, while follow-up MRI demonstrated reduced contrast enhancement and no evidence of locoregional progression.

The development of tympanic membrane ulceration and middle ear mucosal necrosis during follow-up highlights the potential for treatment-related toxicity in tissues involved by the tumor. These findings resolved with conservative management and close clinical surveillance. Given the limited follow-up duration, the present case does not permit conclusions regarding long-term tumor control or delayed radiation-related toxicity. Continued clinical and radiological surveillance is therefore warranted to assess the durability of the observed response and to identify potential late treatment-related complications in this case.
